# Do incentives undermine intrinsic motivation? Increases in intrinsic motivation within an incentive-based intervention for people living with HIV in Tanzania

**DOI:** 10.1371/journal.pone.0196616

**Published:** 2018-06-14

**Authors:** Nancy L. Czaicki, William H. Dow, Prosper F. Njau, Sandra I. McCoy

**Affiliations:** 1 Division of Epidemiology, School of Public Health, University of California Berkeley, Berkeley, CA, United States of America; 2 Health Services and Policy Analysis Graduate Group, School of Public Health, University of California Berkeley, Berkeley, CA, United States of America; 3 Ministry of Health, Community Development, Gender, Elderly, and Children, Dar es Salaam, Tanzania; The Ohio State University, UNITED STATES

## Abstract

**Background:**

Cash and in-kind incentives can improve health outcomes in various settings; however, there is concern that incentives may ‘crowd out’ intrinsic motivation to engage in beneficial behaviors. We examined this hypothesis in a randomized trial of food and cash incentives for people living with HIV infection in Tanzania.

**Methods:**

We analyzed data from 469 individuals randomized to one of three study arms: standard of care, short-term cash transfers, or short-term food assistance. Eligible participants were: 1) ≥18 years old; 2) HIV-infected; 3) food insecure; and 4) initiated antiretroviral therapy (ART) ≤90 days before the study. Food or cash transfers, valued at ~$11 per month and conditional on attending clinic visits, were provided for ≤6 months. Intrinsic motivation was measured at baseline, 6, and 12 months using the autonomous motivation section of the Treatment Self-Regulation Questionnaire (TSRQ). We compared the change in TSRQ score from baseline to 6 and 12 months and the change within study arms.

**Results:**

The mean intrinsic motivation score was 2.79 at baseline (range: 1–3), 2.91 at 6 months (range: 1–3), and 2.95 at 12 months (range: 2–3), which was 6 months after the incentives had ended. Among all patients, the intrinsic motivation score increased by 0.13 points at 6 months (95% CI (0.09, 0.17), Cohen’s d = 0.29) and 0.19 points at 12 months (95% CI (0.14, 0.24), Cohen’s d = 0.49). Intrinsic motivation also increased within each study group at 6 months: 0.15 points in the food arm (95% CI (0.09, 0.21), Cohen’s d = 0.37), 0.11 points in the cash arm (95% CI (0.05, 0.18), Cohen’s d = 0.25), and 0.08 points in the comparison arm (95% CI (-0.03, 0.19), Cohen’s d = 0.21); findings were similar at 12 months. Increases in motivation were statistically similar between arms at 6 and 12 months.

**Conclusion:**

Intrinsic motivation for ART adherence increased significantly both overall and within the food and cash incentive arms, even after the incentive period was over. Increases in motivation did not differ by study group. These results suggest that incentive interventions for treatment adherence should not be withheld due to concerns of crowding out intrinsic motivation.

## Introduction

Financial incentives have been shown to increase a variety of health behaviors and positive health outcomes including health care utilization [1], immunization rates [2], child health status [2], mental health [3], exercise [4], and medication adherence.[5–7] Furthermore, cash transfer programs for poverty alleviation are now standard practice in Latin America and rapidly increasing in Asia and Africa.[8] In the realm of HIV/AIDS, financial incentives can reduce HIV incidence [9–11], increase HIV testing [12–14] and linkage to care after diagnosis [15], and increase antiretroviral therapy (ART) adherence.[5]

Although cash incentives have shown great promise as a public health intervention, it is critical to understand how they achieve their intended effects. Self Determination Theory (SDT), the principal psychological theory of human motivation, is often cited as an explanation of how incentives facilitate behavior change. It distinguishes between intrinsic motivation, which is engaging in an activity because of joy or other positive feelings generated from doing the activity, and extrinsic motivation, which is engaging in an activity because of some separate positive or negative consequence.[16] In the context of incentives, the incentive serves as an extrinsic motivator to engage in the desired behavior. SDT also suggests that under certain conditions where autonomy, competence, and relatedness to others are fostered, extrinsic motivation can be internalized and transformed to intrinsic motivation.[17, 18] Ideally, under this theory, incentives would be provided as a source of external motivation under conditions that facilitate it becoming internalized.

However, some critical of incentives argue that they may ‘crowd out’ intrinsic motivation, making the individual *less* likely to engage in the desired behavior after the incentive period compared to baseline.[19, 20] If true, a consequence is that incentives may have limited durability of effect and could cause harm in the long term.[5, 21] Experimental evidence supporting the ‘crowding out’ hypothesis is largely rooted in the field of psychology, with the most recent studies occurring in education. These studies often occur in laboratory-like, controlled settings where individuals are given a task, such as completing a puzzle or editing papers, and are then given free time.[22] Whether they continue with the task and for how long during free time is compared between a group receiving a reward, monetary or otherwise, and the group not receiving the reward. A meta-analysis of 128 studies found that those receiving the reward spent less time on the task in their free choice time compared to those without the reward.[22–24] However, others contend that incentives do not hamper intrinsic motivation under most conditions, citing the limited generalizability and narrow hypotheses of most experiments.[25, 26]

The crowding out theory has not been explicitly examined in real world settings where the incentivized behavior may be beneficial and improve the individual’s health, as is the case with adherence to HIV treatment or keeping scheduled appointments. For example, many studies on human motivation conducted in education focus on short-term performance-based incentives (e.g., getting a high test score) that are not necessarily directly linked the desired outcome of increasing learning.[19] In addition, contrary to the crowding out theory, some studies have found that incentives can have lasting effects on their intended outcome after the incentive period. For example, although a recent study of conditional cash transfers to increase gym attendance showed a reduction in attendance post-incentive, the level did not drop *below* baseline gym attendance levels nor below that of the control group suggesting that the program did not cause long-term harm (e.g., reduction in intrinsic motivation) or a reduction in the beneficial behavior.[4] Another study found incentives to have long term effects on smoking cessation.[27] These studies did not occur in a laboratory setting and are likely a more accurate representation of incentive response and motivation in a real-world setting.

The few studies examining the effect of incentives on adherence to ART have targeted populations with poor adherence in the U.S.[5] While all noted an increase in adherence or decrease in viral load during follow-up, in the few studies that measured post-incentive outcomes, adherence levels returned to baseline after the intervention period, suggesting lack of durability of the incentives’ effect.[5] However, no study has empirically examined whether incentives ‘crowd out’ intrinsic motivation in the context of HIV treatment adherence. For example, if incentives crowd out intrinsic motivation, implementers might need to be judicious about when and among whom to use incentives to bolster engagement in a health behavior. However, if there is no evidence for crowding out, then the scale-up of successful incentive-based interventions should not be hampered by this concern. To address this question, we had the opportunity to examine whether crowding out occurred in a study of conditional food and cash incentives in Tanzania using an empirical measure of intrinsic motivation.[28] We aimed to explore whether levels of internal motivation changed between baseline and after participants have received up to six food or cash transfers, and whether this difference varied by study arm. Our goal was to understand how receiving a financial incentive may impact intrinsic motivation in a real-world, resource-constrained setting.

## Methods

### Population and setting

We conducted a secondary analysis of data from a randomized controlled trial (clinicaltrials.gov: NCT01957917) evaluating the impact of food and cash transfers on adherence among HIV-positive adults in Shinyanga, Tanzania.[29] Participants were recruited from two government hospitals and one government health clinic in Shinyanga region, Tanzania. The facilities were located in peri-urban areas and served patients who both live in town and in distant rural areas. Patients pick up their medication directly from the clinic on the same day as their clinic visits, thus in this setting, attending clinic visits is a precursor to medication adherence.

In the parent study, eligible patients met the following inclusion criteria at the time of enrollment: 1) ≥18 years of age; 2) living with HIV infection; 3) initiated ART ≤90 days before enrollment; and 4) food insecure, as measured by the Household Hunger Scale.[30, 31] Participants who were severely underweight (BMI<16.0) were excluded from the study, as these individuals required therapeutic food support (ready-to-use food products) for nutritional recovery. In total, 805 patients were recruited and randomized in a 1:3:3 ratio into one of 3 arms: nutritional assessment and counseling (NAC; comparison group), NAC plus monthly food transfer, or NAC plus cash transfer (hereafter, ‘transfers’ is used interchangeably with ‘incentives’). The parent trial was powered using a non-inferiority design to test the primary hypothesis that cash assistance was at least as effective as food assistance at improving ART adherence.[29] The secondary objective was to determine whether ART adherence among those offered food or cash transfers (combined) was better than those receiving the standard of care. Thus, following sample size determination procedures for what is often referred to as the “three-arm ‘gold standard’ non-inferiority design”, the appropriate allocation ratio for the study was determined to be 1:3:3.[32, 33] The food and cash incentives were of equal value, and patients in the food or cash arms were eligible for up to 6 consecutive monthly transfers conditional on attending routine clinic appointments (within a +/-4 day window). At baseline, 6, and 12 months, in-person surveys were administered at the clinic by trained research assistants in Kiswahili and data were extracted from patients’ medical files.

This analysis excluded participants missing internal motivation data at baseline (n = 6), those missing both the 6-month and 12-month surveys (n = 174), and those who started ART on the day of enrollment (n = 156), as they were not asked questions regarding intrinsic motivation to take ART. Those that completed the baseline survey and either the 6-month survey and/or 12-month survey were eligible for inclusion (n = 469).

### Measurements

#### Primary outcome

Since actual behavior (i.e., adherence to ART or retention in care) is subject to many barriers, often outside of the patient’s control, this observable behavior was not an adequate measure of intrinsic motivation. For example, someone may not be able to attend clinic and pick up medication due to cost or distance, even if they are highly motivated to attend.[34–38] Thus, intrinsic motivation was measured at baseline (before incentives), 6 months (after completion of incentive period), and 12 months (6 months post-incentive) using the autonomous motivation section of the Treatment Self-Regulation Questionnaire (TSRQ), originally designed to assess motivations for remaining in a weight loss program [28]. The TSRQ was modeled after self-regulation questionnaires by Ryan and Connell [39], and is based on Self-Determination Theory.[16] The autonomous section of this scale measures the extent to which individuals choose to engage with a specific health behavior because of its importance to them (intrinsic motivation), rather than its importance to others or in response to external stimuli (extrinsic motivation). The TSRQ has been used to examine the relationship between intrinsic motivation and weight loss maintenance and exercise [28], smoking cessation, glucose control among patients with diabetes [40], adherence to medication for chronic health conditions [41], and adherence to ART.[42] It has also been further validated in the contexts of tobacco use, diet and exercise.[43] We piloted the scale in Kiswahili to ensure questions were interpreted as intended. Participants who were on ART at baseline rated their level of agreement to statements describing reasons they may take their HIV medication as prescribed using a 3 point Likert Scale (1 = *not at all true*, 2 = *somewhat true*, 3 = *very true*). For example, participants were asked, “The reason you take your HIV medication as it was prescribed to you is because taking your HIV medication is consistent with your life goals” (other questions are included in S1 Table). Consistent with previous studies [28, 42–44], the measure of intrinsic motivation was defined as the average score across the five statements. Change in motivation from baseline to 6 months was defined as the difference in score between the 6-month survey and baseline. Likewise, change in motivation at 12 months was defined as the difference in score between the 12-month survey and baseline.

#### Exposure

We aimed to determine if intrinsic motivation changed between baseline and the post-transfer period and whether that difference varied between study groups. Thus, the primary exposure was the randomly assigned study arm: food transfers, cash transfers, or comparison group.

#### Covariates

We examined heterogeneity in the change in motivation by additional factors representing determinants of intrinsic motivation as theorized by SDT. Deci and Ryan suggest that factors that enhance competence, autonomy, or relatedness will enhance intrinsic motivation and, conversely, factors that undermine these will decrease intrinsic motivation.[18] Additionally, Williams linked autonomous motivation, that coming from within, with self-rated current health, severity of illness, and perceived barriers in an investigation of the relationship between motivation and medication adherence.[41] Factors that control behavior, like rigid social roles or inability to make decisions about your own behavior, may also undermine autonomous self-regulation. With this in mind, we proposed to examine heterogeneity of change in motivation by the following covariates: age, gender, head of household status, marital status, education, religion, working status, number of people in the household, food insecurity (according to the Household Hunger Survey) [30], whether or not the individual has children, inability to go to school or work due to illness, role in decision making regarding own health care, self-rated health (1–10 scale), and barriers to care. All factors were self-reported in the survey at baseline (see S1 Table). We created an index of barriers to care that was the sum of responses (0 = *not a problem*, 1 = *somewhat of a problem*, 2 = *big problem*) to questions about 11 barriers to medical advice or treatment (e.g., getting permission to go or getting money needed for treatment).

### Analysis

To determine whether intrinsic motivation changed between baseline and post-transfer periods and whether that change was different across arms, we conducted paired t-tests among the total study population (pooled across study arms) and also within arms. Effect size was expressed as Cohen’s d statistic for each test. Cohen’s d is a standardized measurement of the difference between two means and is calculated as difference in means divided by the standard deviation. In general, an effect of 0.2 is considered small, 0.5 medium, and above 0.8, large.[45] We compared the change in intrinsic motivation between arms using linear regression. These same methods were repeated to compare the change in motivation between baseline and 12 months. The results of an analysis limited to the subset of 247 participants with complete intrinsic motivation data at baseline, 6 months, and 12 months were identical to the analysis with all patients; for brevity, we present the analysis of the larger group of patients in this manuscript.

To examine heterogeneity across potential determinants of intrinsic motivation, we added the variable of interest and the two-way interaction term between the determinant and arm to the model. The Liu method was used to control false discovery rate in order to account for multiple testing in this subgroup analysis (*multproc* package in Stata).[46] To examine the impact of loss to follow-up (197 participants did not complete the 6 month survey), we repeated the analysis with inverse probability of censoring weights (IPCW) to account for differential attrition. Probability of censoring was predicted using pooled logistic regression and included arm, age, food insecurity status at baseline, education, clinic, baseline working status, sex, marital status, and barrier score as main-term predictors. All analysis was conducted in Stata version 13 (College Station, TX).

### Ethics, consent and permissions

The Tanzanian National Institute for Medical Research and the Committee for Protection of Human Subjects at the University of California, Berkeley approved this study. Informed consent was obtained from all individual participants included in the study.

## Results

**Fig 1:**
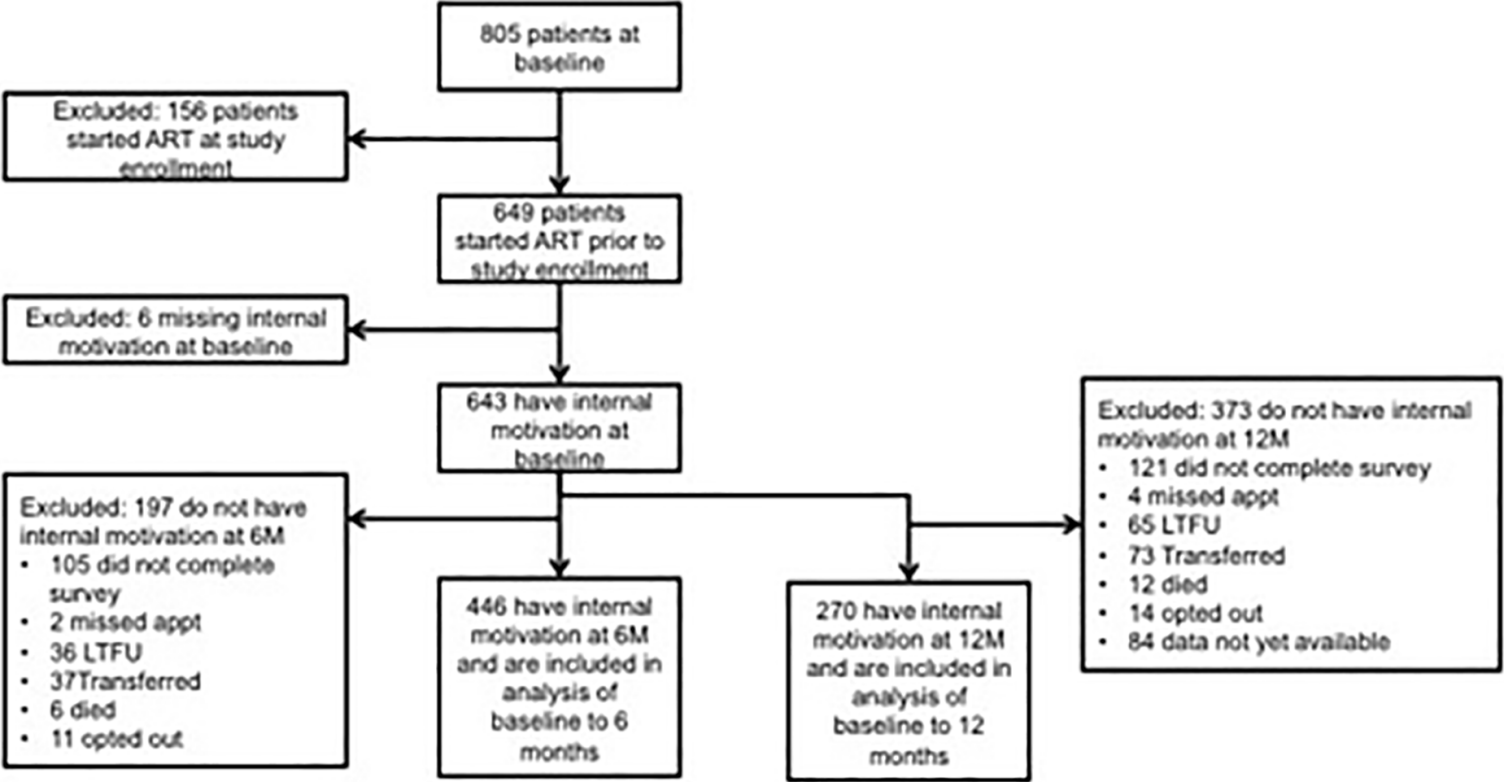
Flow diagram of participants included and excluded from analysis.

Overall, 469 participants were included in the analysis, of whom 446 completed both the baseline and 6-month surveys (N = 195 food arm; N = 207 cash arm, N = 45 comparison arm) and 270 completed the baseline and 12 month surveys (Fig 1). The average age was 37 years, 67% were female, 27% had no formal education, 89% had children, 76% were Christian, and 38% were married (Table 1). At baseline, the majority of participants were currently working (61%) and 60% were head of household. Average self-rated health at baseline was 8.25 out of 10, although 56% of participants had been unable to work or attend school due to illness in the past 12 months.

**Table 1:** Characteristics of participants completing both baseline and 6-month surveys, Tanzania, 2014–2015.

Characteristic	N	(%)
Total	446	(100)
Study Arm		
Food transfers	194	(44)
Cash transfers	207	(46)
Comparison	45	(10)
Female	299	(67)
Education		
None	122	(27)
Any	324	(73)
Religion		
Christian	340	(76)
Islam	77	(17)
None	29	(7)
Marital Status		
Single	64	(14)
Married	172	(38)
Unmarried with partner	18	(4)
Divorced/Widowed/Separated	192	(44)
Currently Working	272	(61)
Head of Household	268	(60)
Who has the final say on decisions about how or when you obtain your own healthcare		
You alone	320	(72)
Your partner/spouse	29	(7)
Someone else alone	11	(3)
You jointly	85	(19)
Baseline Household Hunger Scale (HHS)		
Moderate hunger	266	(60)
Severe hunger	180	(40)
6-month HHS		
Little to no hunger	160	(39)
Moderate hunger	210	(50)
Severe hunger	46	(11)
Has children	394	(89)
Ever unable to attend work or school due to illness in the last 12 months	248	(56)
	**Mean**	**(SD)**
Age (years)	37.07	(10.43)
Number in household	3.74	(2.08)
Self-rated health (1–10 scale)	8.25	(1.46)
Barriers to care (max 22)	2.53	(2.04)
Internal motivation at baseline	2.79	(0.36)
Internal motivation at 6M	2.91	(0.23)

**Fig 2:**
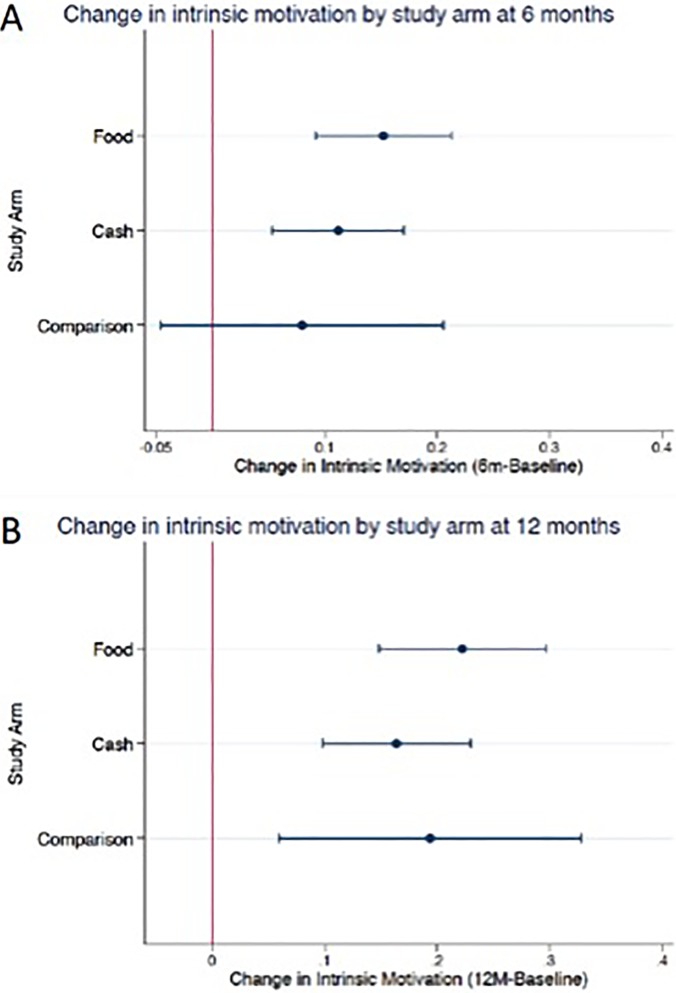
**Mean change in intrinsic motivation by study arm with 95% confidence intervals at 6 months (A) (N = 446) and 12 months (B) (N = 270)^ab^** ^a^The red line at 0 indicates no change in intrinsic motivation. ^b^The mean change in intrinsic motivation is presented along with the estimated 95% confidence interval.

The mean intrinsic motivation score was 2.79 at baseline (range: 1–3), 2.91 (range: 1–3) at 6-months, which was after the opportunity to receive food or cash transfers, and 2.95 at 12 months (range: 2–3), which was 6 months after incentives had ended. The level of intrinsic motivation significantly increased overall over both time periods and within each study group (Table 2). Among all patients, the intrinsic motivation score increased by 0.13 points at 6 months (95% CI (0.09, 0.17), Cohen’s d = 0.29) and 0.19 points at 12 months (95% CI (0.14, 0.24), Cohen’s d = 0.49). We also observed increases in intrinsic motivation in each incentive arm. The increase in intrinsic motivation at 6 months was 0.15 points in the food arm (95% CI (0.09, 0.21), Cohen’s d = 0.37), 0.11 points in the cash arm (95% CI (0.05, 0.18), Cohen’s d = 0.25), and 0.08 points in the comparison arm (95% CI (-0.03, 0.19), Cohen’s d = 0.21) (Fig 2A). At 12 months, the increase in motivation from baseline was 0.22 points in the food arm (95% CI (0.14, 0.31), Cohen’s d = 0.52), 0.16 points in the cash arm (95% CI (0.10, 0.22), Cohen’s d = 0.47), and 0.19 points in the comparison arm (95% CI (0.05, 0.34), Cohen’s d = 0.49) (Fig 2B). The change in motivation did not differ significantly by arm at either time point, although qualitatively, the increase in motivation was slightly greater in the food group. For example, at 6 months the change in motivation in the food arm was 0.07 points greater than that of the comparison (95% CI: -0.07, 0.21), and the change in the cash arm was 0.03 points greater than that of the comparison (95% CI: -0.11, 0.17) (Table 3). Weighting the models by the inverse probability of censoring did not significantly change the results (Table 3).

**Table 2:** Paired T-tests comparing intrinsic motivation scores at 6 and 12 months to baseline^a^.

**Comparison of 6-month values to baseline (N = 446)**																
** **			**N**	**Baseline**		**6-Month**		**Difference (6M-baseline)**		**SD**			**95% CI**		** **	**Cohen's d**
Overall			446	2.79		2.91		0.13		0.43			(0.09, 0.17)		**	0.29
Food Transfer Arm			194	2.77		2.92		0.15		0.42			(0.09, 0.21)		**	0.37
Cash Transfer Arm			207	2.80		2.91		0.11		0.45			(0.05, 0.18)		**	0.25
Comparison Arm			45	2.79		2.87		0.08		0.38			(-0.03, 0.19)			0.21
**Comparison of 12-month values to baseline (N = 270)**																
			**N**	**Baseline**		**12-Month**		**Difference (12M-baseline)**		**SD**			**95% CI**		** **	**Cohen's d**
Overall			270	2.76		2.95		0.19		0.39			(0.14, 0.24)		**	0.49
Food Transfer Arm			104	2.72		2.94		0.22		0.43			(0.14, 0.31)		**	0.52
Cash Transfer Arm			134	2.79		2.95		0.16		0.35			(0.10, 0.22)		**	0.47
Comparison Arm			32	2.76		2.95		0.19		0.39			(0.05, 0.34)		*	0.49

*p<0.05

**p<0.001

a. Intrinsic motivation was the average score of a 3 point Likert scale on 5 questions from the autonomous motivation scale of the TSRQ.

Note: Baseline values for the 6 and 12 month comparisons are different due to different sample size and different individuals included in each analysis

**Table 3:** Linear regression model of change in intrinsic motivation across study arm.

**Change in intrinsic motivation (6M-baseline) (N = 446)**						
	Unadjusted^a^			Adjusted^b^		
Group	Coefficient	95% CI	p-value	Coefficient	95% CI	p-value
Comparison	Ref		0.479	Ref		0.375*
Food Transfers	0.070	(-0.07, 0.21)	0.308	0.050	(-0.02, 0.13)	0.162
Cash Transfers	0.030	(-0.11, 0.17)	0.650	0.040	(-0.03, 0.11)	0.277
**Identical models with inverse probability of censoring weights**						
	Unadjusted^a^			Adjusted^b^		
Group	Coefficient	95% CI	p-value	Coefficient	95% CI	p-value
Comparison	Ref		0.430	Ref		0.573*
Food Transfers	0.07	(-0.05, 0.19)	0.271	0.04	(-0.03, 0.11)	0.300
Cash Transfers	0.02	(-0.10, 0.14)	0.752	0.03	(-0.05, 0.10)	0.465
**Change in intrinsic motivation (12M-baseline) (N = 270)**						
	Unadjusted^a^			Adjusted^b^		
Group	Coefficient	95% CI	p-value	Coefficient	95% CI	p-value
Comparison	Ref		0.508	Ref		0.842*
Food Transfers	0.03	(-0.12, 0.18)	0.710	-0.01	(-0.07, 0.05)	0.767
Cash Transfers	-0.03	(-0.18, 0.12)	0.698	0.002	(-0.06, 0.07)	0.932
**Identical models with inverse probability of censoring weights**						
	Unadjusted^a^			Adjusted^b^		
Group	Coefficient	95% CI	p-value	Coefficient	95% CI	p-value
Comparison	Ref		0.364			0.829*
Food Transfers	0.03	(-0.12, 0.18)	0.682	-0.01	(-0.08, 0.06)	0.811
Cash Transfers	-0.04	(-0.18, 0.10)	0.594	0.003	(-0.06, 0.07)	0.923

a. Model only contains study arm

b. Adjusted for clinic, sex, age, education, baseline intrinsic motivation, and baseline food insecurity (HHS category)

*Wald test for equivalence of all arms

Analysis of heterogeneity revealed no differences in change in motivation within subgroups. Initially, only age appeared to significantly modify the effect of study arm (p = 0.05), but after adjusting for multiple comparisons, this was no longer significant (critical p-value = 0.002).

## Discussion

We examined changes in intrinsic motivation in a trial of food and cash incentives for HIV treatment adherence in Shinyanga, Tanzania. We found that intrinsic motivation to take ART significantly increased both overall and within the food and cash incentive arms between baseline and the end of the transfer period and 6-months after the incentive period. We found no difference in the size of the increase by study arm and there was no evidence of heterogeneity by proposed determinants. To our knowledge, this study is the first to empirically examine the crowding out hypothesis regarding incentives for HIV treatment in a real-world, resource-limited setting, and we found no evidence to support this theory.

The idea that incentives can undermine intrinsic motivation has historically been an argument against their use.[20–22] Empirical data from this sample in Tanzania shows, contrary to the crowding out hypothesis, that intrinsic motivation is higher post-incentive compared to baseline. This relationship was robust to both adjustment for patient characteristics and inverse probability of censoring weighting to account for loss to follow-up. It also held for both 6 and 12 months of follow-up. In addition to the quantitative data presented here, qualitative data from in-depth interviews suggests that participants used the incentives to both overcome economic constraints and offset opportunity costs to attending clinic.[47] Furthermore, these results are consistent with the primary results from the main study, had we only used the end behavior as the motivation measure.[48] Both retention and adherence, measured using the medication possession ratio, was higher among the food and cash groups at 6 months compared to the comparison group and this relationship held for the cash group at 12 months (6 months after the incentive ended). This suggests that the transfer is not merely an extrinsic motivator, and in fact, that it may have little impact on motivation to attend clinic and instead overcame financial barriers.

When considering incentives to engage in a health behavior, there is likely a difference between offering an economic incentive to someone who is economically constrained, whereby the incentive makes the behavior possible by lifting the constraint, and offering an incentive to someone with sufficient resources whereby the incentive may provide additional motivation to engage in the behavior.[49] The context and the ‘signal’ sent by the incentive (e.g., beliefs about the target behavior) likely plays an important role in how incentives impact motivation.[20] This particular intervention targeted a vulnerable population of people living with HIV and was designed to address food insecurity and overcome economic constraints for ART treatment, such as reductions in employment and productivity often experienced prior to and immediately after starting ART.[50, 51] The incentive was valued to be consistent with cash transfers provided through the Tanzania Social Action Fund (a government-run anti-poverty program) to avoid coercive effects and the program was designed to be short term. Even though the baseline scores of intrinsic motivation were close to the maximum score possible, we still observed significant increases after the conclusion of the intervention period. While we cannot definitively refute the crowding out hypothesis in all arenas, our results suggest that this incentive intervention in this context likely did not *reduce* the reported level of intrinsic motivation for treatment adherence among HIV-infected adults. It is possible that the crowding out hypothesis applies differentially to situations where the incentive lifts a budgetary constraint for a behavior in which the individual is highly motivated to engage (e.g., ART), compared to those situations where the incentive primarily operates as an extrinsic motivator, a nuance that has been examined in the literature on incentives and prosocial behavior.[20, 52] For example, some have hypothesized that incentives and altruism can be complimentary (‘crowding in’ motivation) or substitutes (‘crowding out’ motivation), depending on the specific context.[20] Thus, understanding the specific factors that influence whether incentives enhance or undermine intrinsic motivation in the HIV context is worthy of further study.There are several limitations to this analysis. First, motivation was measured by the TSRQ scale, which was selected in order to build on previous research on intrinsic motivation for treatment adherence. However, use of this scale in low-income countries is limited. Although the scale has been validated for many health outcomes in myriad settings and questions were reviewed, vetted, and piloted with local staff and translated appropriately, it was not directly validated in this setting. Observed behavioral data, such as clinic attendance after the transfer period is over, is an alternative indicator of intrinsic motivation. However, observed behavior is influenced by multiple drivers *in addition to* intrinsic motivation, such as ability to access care, experience with providers, illness, migration, and other socioeconomic and psychosocial factors. Thus, it would be difficult to fully equate observed behavior with intrinsic motivation, and this is why studying intrinsic motivation is valuable even if behavioral data are available. Nevertheless, it is reassuring that our findings on intrinsic motivation are consistent with objective measures of behavior, as both adherence and retention were higher in the incentive groups at 6 months and this relationship held for the cash group at 12 months.[48] Thus, given its limitations, the TSRQ scale was the best-suited measurement tool for this application at this time.

Secondly, since the baseline scores on the TSRQ were already rather high on the scale, there may be ceiling effects, where the participants are unable to score any higher on the scale. This may be partially due to our use of a 3-item Likert scale, whereas others have used 5- or 7-point scales.[28, 42] This may limit our ability to test whether external incentives were internalized in the form of increased intrinsic motivation since some in the transfer arms could not possibly score higher. Furthermore, this was a secondary analysis of data from a randomized trial that was not powered to detect a change in motivation, nor heterogeneity by subgroup. Although the increase in motivation was statistically significant overall and within both the food and cash arms, we cannot rule out lack of power as the reason for the non-significant increase in the comparison arm and non-significant differences between groups. Similarly, lack of power may explain why we did not detect significant heterogeneity among subgroups. Lastly, despite these promising findings, we cannot completely rule out the possibility that some crowding out is occurring. For example, it is possible that intrinsic motivation naturally increases throughout treatment and a positive overall change in motivation could occur despite some negative ‘crowding-out’ effects from the incentives.

This analysis also had several strengths, the most significant is that it was done within the context of a randomized trial, which limited confounding by unmeasured factors. Secondly, the intervention and measurements were standardized and implemented in the same manner across arms. The trial design also allowed us to measure intrinsic motivation at baseline, immediately at the conclusion of the incentive period, and also 6-months after the incentives had ceased providing a clear comparison of motivation levels and outcomes, something often missing from other incentive studies. Furthermore, the TSRQ was developed in line with self-determination theory, the underlying theory of the crowding out hypothesis. Using the sub-scale of this widely used tool increases comparability of our results to other studies. This scale has been used with many health outcomes and has been validated across settings in the developed world. Of note, the distribution of responses in our study (baseline intrinsic motivation 2.79 out of 3; 6-month: 2.91) is similar to other studies that have used this instrument. In the first use of the TSRQ autonomous scale used in a weight loss study, the mean was 13.8 out of 15 [28]; in a review of studies on smoking and/or diet and exercise 3 of 4 sites had a mean of roughly 6 out of 7 [43]; in a study of patients with heart failure, nearly half of participants had the maximum score of 7 [44]; and in a study specifically looking at ART use, the mean TSRQ score for autonomous motivation was 6.5 out of 7 [42]. Lastly, the results of the sensitivity analysis using IPCW to account for loss to follow-up were consistent with the unadjusted and adjusted results of complete cases, which demonstrates the robustness of our results.

In conclusion, this is the first study to empirically examine the crowding out hypothesis in the context of an incentive intervention in a resource-limited setting. Further research is needed to explore this relationship in different settings and with incentives of different amount and duration. Given these results, incentive interventions for treatment adherence should not be withheld due to concerns of crowding out intrinsic motivation.

## Supporting information

S1 Table Distribution of answers to each question in the TSRQ sub-scale at each time point. (DOCX)
