# Supplementary material for: Do incentives undermine intrinsic motivation? Increases in intrinsic motivation within an incentive-based intervention for people living with HIV in Tanzania
Source: PLoS One. 2018 Jun 14;13(6):e0196616. doi: 10.1371/journal.pone.0196616 (PMC6002082; doi:10.1371/journal.pone.0196616)
Supplement: S1 Table — (DOCX) [file pone.0196616.s001.docx]

**S1 Table. Distribution of answers to each question in the TSRQ sub-scale at each time point**

|  | Baseline  (N =643) | | 6-months  (N =446) | | 12 months  (N =270) | |
| --- | --- | --- | --- | --- | --- | --- |
| Item | Mean | (SD) | Mean | (SD) | Mean | (SD) |
| **The reason you take your HIV medication as it was prescribed to you is…** |  |  |  |  |  |  |
| Because you feel that you want to take responsibility for your own health | 2.76 | (0.46) | 2.93 | (0.29) | 2.97 | (0.18) |
| Because you have carefully thought about it and believe it is very important for many aspects of your life | 2.81 | (0.47) | 2.91 | (0.31) | 2.94 | (0.24) |
| Because taking your HIV medication is consistent with your life goals | 2.78 | (0.50) | 2.89 | (0.36) | 2.93 | (0.30) |
| Because you personally believe it is the best thing for your health | 2.83 | (0.46) | 2.91 | (0.34) | 2.95 | (0.21) |
| Because it is an important choice you really want to make | 2.79 | (0.50) | 2.93 | (0.30) | 2.96 | (0.22) |
